# Cost–effectiveness of pancreatic cancer screening: Time for a more tailored approach

**DOI:** 10.1002/ueg2.12380

**Published:** 2023-03-20

**Authors:** Livia Archibugi, Gabriele Capurso, Marcia Irene Canto

**Affiliations:** ^1^ Pancreato‐Biliary Endoscopy and Endosonography Division Pancreas Translational & Clinical Research Center San Raffaele Scientific Institute IRCCS Vita‐Salute San Raffaele University Milan Italy; ^2^ Division of Gastroenterology Department of Medicine Johns Hopkins University School of Medicine Baltimore Maryland USA

**Keywords:** cancer, Endoscopic Ultrasound, magnetic resonance imaging, pancreatic cancer, screening

Despite some progress, pancreatic adenocarcinoma (PDAC) remains a deadly disease with a dismal prognosis.^1^ Better tools to prevent, diagnose earlier and treat PDAC have been advocated, and all represent unmet needs.^2^

In the past 20 years, the idea that secondary prevention (surveillance) could allow early detection in well‐defined high‐risk individuals (HRIs) based on their family history alone (familiar pancreatic cancer (FPC)) or on the presence of specific germline mutations has emerged. However, this was considered to be indicated as part of research protocols.^3^ According to the WHO, screening programs should accomplish certain criteria defined by Wilson & Jungner in the late 60s.^4^ As for PDAC screening, many of these criteria have remained open questions for decades; among them: a) is there an accepted treatment for patients with recognized disease? b) is there a recognizable latent or early symptomatic stage of the disease? c) is the natural history of the condition adequately understood? d) is the process cost‐effective?

In the current issue of United European Gastroenterology Journal,^5^ Ibrahim and colleagues report data on the cost‐effectiveness of PDAC surveillance in a large cohort of 347 CDKN2A mutation carriers, a cohort of patients with an extremely high lifetime risk of developing PDAC (Relative Risk reported to be between 13 and 39).^6^ The authors have previously reported that surveillance in these subjects leads to PDAC diagnosis in 9% during a median follow‐up of 5.6 years, with 71% of diagnosed PDAC being resected.^7^ This approach results in a 12% decrease in mortality with a life expectancy increase exceeding 2 years. These results are found to be cost‐effective with a cost‐utility ratio of 14,000 euro per quality‐adjusted life year gained. The authors conclude that when the lifetime risk of PDAC is >10%, screening is cost‐effective.

These findings are encouraging and suggest that surveillance may be justified in CDKN2A mutation carriers and other groups of individuals with moderate to high estimated lifetime risk, such as patients with Peutz‐Jegher syndrome. Additional important findings come from the multicenter Cancer of Pancreas Screening‐5 (CAPS 1–5) studies conducted over the past 25 years in the United States^8^ that reported long‐term results of surveillance of >1700 HRIs with 19 surveillance‐detected PDACs of whom 70% had resectable disease (58% stage I). Notably, the median survival for patients with surveillance‐detected PDAC was 9.8 years, that is radically different from the median survival of 1.5 years for HRIs with PDAC detected outside surveillance.^8^ Despite a relatively small number of detected PDACs, a 5‐year survival rate of 73% in the CAPS 1–5 cohort underscores the potential large impact of surveillance in highly selected individuals with a familial or genetic predisposition. The question is what to do for the majority of average risk individuals, as population‐based screening is not feasible.

While both the CAPS5 and Leiden data shed some optimism showing that early diagnosis is possible and can lead to high rates of resectable disease and improved survival, there are important differences to notice and grey areas to be explored to refine surveillance strategies in a personalized manner. First, the rate of PDAC diagnosed in CAPS is much lower, probably due to the lower rate (48%) of individuals with a pathogenic germline variant and only 4.7% with a CDKN2A mutation. Indeed, the yield of surveillance is known to be much lower in FPCs, especially when they do not carry pathogenic mutations^9^, ^10^ and its cost‐effectiveness is questionable.

Another grey area concerns the frequency of surveillance, as currently an annual interval is proposed for all HRIs, except for those with pancreatic abnormalities requiring a more intensive follow‐up. In the paper by Ibrahim et al.,^5^ a shorter screening interval was also taken into consideration. One of the possible bi‐annual programs would lead to an increased rate of resectability (up to 90%), yet being cost‐effective only for patients with a lifetime PDAC risk >32%, a rate that may only apply to subjects with Peutz‐Jeghers syndrome or some form of hereditary chronic pancreatitis.

The Leiden group has started a bi‐annual surveillance with MRI alternated with EUS every 6 months in CDKN2A mutation carriers, but whether this more intensive screening has more favourable results, has not been published yet.

In this context, we believe that future studies should focus on evaluating the precise lifetime risk of HRIs based not only on their germline variant but also on additional risk modifiers, such as age, smoking, and family history, in order to lead to a more tailored approach. An example of a more bespoke approach that defines screening intervals based on the individual syndrome and scoring systems is being taken into consideration for breast cancer^11^ and polyposis syndromes.^12^ Ultimately, a precision medicine approach to the surveillance and treatment of detected PDAC considering individual differences in patients' genes, environment, and lifestyle would be ideal.

The adoption of EUS or MRI as a surveillance method is another open question for pancreatic surveillance in HRI, with some preliminary data showing a diagnostic yield similar to MRI for target lesions^9^ but a possible better yield for EUS in detecting solid lesions.^9^, ^10^ The use of index EUS as a PDAC screening method has been evaluated in a single study^13^ showing its possible cost‐effectiveness, especially for HRI at a high‐lifetime risk of PDAC (>10.8%). Similar findings were reported by Corral et al. who found EUS to be the most cost‐effective surveillance strategy for subjects with the highest risk,^14^ such as Peutz‐Jeghers, hereditary pancreatitis, and FPC with ≥3 first‐degree PDAC relatives, but more solid data on the topic are needed.

In the “research agenda” of PDAC screening, one last big open question is the relevance of biomarkers, whether blood‐based or directly evaluated in the pancreatic juice.^15^ Despite many efforts being done in identifying a possible predictive biomarker, this particular field is probably the one lacking the most when it comes to solid data proving its potential.

As it is likely that many factors concur in a non‐linear, complex, and somehow unpredictable manner to determine the actual risk of developing PDAC in HRIs, the advent of more sophisticated prediction models such as the employment of Artificial Intelligence is possibly going to affect the field of PDAC screening in the near future.

In conclusion, the study by Ibrahim et al. found pancreatic cancer screening to be cost‐effective in patients with CDKN2A mutations. Nonetheless, this assumption cannot be extended to all HRIs and the cost‐effectiveness should be evaluated separately for carriers of each mutation increasing the risk in order to tailor the surveillance. As for FPCs with negative extensive genetic investigations, more data are needed to prove that surveillance is indicated and cost‐effective.

## CONFLICTS OF INTEREST STATEMENT

The authors have no conflicts of interest to declare.

## Data Availability

Data sharing is not applicable to this article as no new data were created or analyzed in this study.
